# The Solvability of the Discrete Boundary Value Problem on the Half-Line

**DOI:** 10.3390/e23111526

**Published:** 2021-11-17

**Authors:** Magdalena Nockowska-Rosiak

**Affiliations:** Institute of Mathematics, Lodz University of Technology, ul. Politechniki 8, 93-590 Łódź, Poland; magdalena.nockowska@p.lodz.pl

**Keywords:** discrete boundary value problem on infinite interval, fixed-point theorem, Fredholm operator of index 0, perturbation technique, 39A22, 47H10, 34B40

## Abstract

This paper provides conditions for the existence of a solution to the second-order nonlinear boundary value problem on the half-line of the form $\Delta \left(a(n)\Delta x(n)\right)=f\left(n+1,x\left(n+1\right),\Delta x\left(n+1\right)\right),\;n\in N\cup \left\{0\right\},$ with αx(0)+βa(0)Δx(0)=0,x(∞)=d, where d,α,β∈R, $\alpha^{2}+\beta^{2}>0$. To achieve our goal, we use Schauder’s fixed-point theorem and the perturbation technique for a Fredholm operator of index 0. Moreover, we construct the necessary condition for the existence of a solution to the considered problem.

## 1. Introduction

In various physical areas, such as hydrodynamics or the unsteady flow of gas through a semi-infinite porous media, studying radially symmetric solutions leads to the Sturm–Liouville equation with boundary value conditions of the form $x^{\prime }\left(0\right)=0$, x(∞)=C, C∈(0,1); see for example [1,2]. Let us remind the reader of the classical Sturm–Liouville boundary value problem on the half-line:

(1) $$\left\{\begin{array}{c} {\left(px^{\prime }\right)}^{\prime }\left(t\right)+\lambda \phi \left(t\right)f\left(t,x\left(t\right)\right)=0,\;t\in \left(0,\infty \right)\\ \alpha_{1}x\left(0\right)-\beta_{1}\lim_{t\to 0^{+}}p\left(t\right)x^{\prime }\left(t\right)=0\\ \alpha_{2}\lim_{t\to \infty }x\left(t\right)+\beta_{2}\lim_{t\to \infty }p\left(t\right)x^{\prime }\left(t\right)=0, \end{array}\right.$$

where λ>0, f:[0,∞)×R→R is continuous, ϕ:(0,∞)→∞ is continuous, $p\in C\left[0,\infty \right)\cap C^{1}\left(0,\infty \right)$, p>0 on (0,∞), $\int_{0}^{\infty }\frac{ds}{p(s)}<\infty$, $\alpha_{i},$$\beta_{i}\geq 0$, i=1,2, $\alpha_{2}\beta_{1}+\alpha_{1}\beta_{2}+\alpha_{1}\alpha_{2}\int_{0}^{\infty }\frac{ds}{p(s)}>0$. Many authors considered the above problem or its simplifications, see, for instance, [3,4,5,6,7] or slightly different boundary value problems on the half-line [8,9,10,11] and the references therein.

Difference equations represent the discrete counterpart of ordinary differential equations and are usually studied in connection with the numerical analysis. In this paper we consider the following discrete boundary value problem on the half-line:

(2) $$\left\{\begin{array}{c} \Delta \left(a(n)\Delta x(n)\right)=f\left(n+1,x\left(n+1\right),\Delta x\left(n+1\right)\right),\;n\in N\cup \left\{0\right\},\\ \alpha x(0)+\beta a(0)\Delta x(0)=0,\;x(\infty )=d, \end{array}\right.$$

where $x\left(\infty \right)=\lim_{n\to \infty }x\left(n\right)$, d∈R, α,β∈R, $\alpha^{2}+\beta^{2}>0$. We want to construct sufficient conditions for the existence of a solution to (2) in dependence on the parameters α, β. First, we divide our consideration into two cases, when problem (2) is without resonance, which means that $\beta \neq \alpha \sum_{l=0}^{\infty }\frac{1}{a(l)}$, and with resonance. For the problem without resonance we use the fixed-point approach, which requires $\sum_{l=0}^{\infty }\frac{l}{a(l)}<\infty$ and the growth condition on a nonlinear continuous function *f*. In both cases, with and without resonance, we prove that $\sum_{l=0}^{\infty }\frac{l}{a(l)}<\infty$ is the necessary condition for the existence of a solution to (2). By the resonant case we mean the following problem:

$$\left\{\begin{array}{c} \Delta \left(a(n)\Delta x(n)\right)=f\left(n+1,x\left(n+1\right),\Delta x\left(n+1\right)\right),\;n\in N\cup \left\{0\right\},\\ x\left(0\right)+a\left(0\right)\left(\sum_{l=0}^{\infty }\frac{1}{a(l)}\right)\Delta x\left(0\right)=0,\;x\left(\infty \right)=d. \end{array}\right.$$

This is called resonant, because for d=0 we can write the above problem in an abstract form, Lx=Nx, where *L* is a linear, noninvertible operator, and *N* is a nonlinear operator. We notice that the noninvertible operator *L* is a Fredholm operator of index 0 and to obtain a solution to the above problem we use the Przeradzki perturbation method, see [12,13]. We construct Landesmann–Lazer type conditions for a bounded and continuous nonlinear function *f*. The used perturbation technique allows us to establish sufficient conditions for the existence of a solution to the above problem not only for d=0, but for all d∈R. The Przeradzki perturbation method is one of the tools used to deal with boundary value problems in the resonant case. Another classical approach is Mawhin’s coincidence degree, see for example [14]. More information about properties of Fredholm operators can be found in [13,15,16] and the references therein.

Many authors have considered a discrete version of (1) or its generalizations using different tools, see for example [2,17,18,19,20,21] and the references therein. Lian et al., in [19], established the sufficient conditions for the existence of one and three solutions of the following problem:

$$\left\{\begin{array}{c} -\Delta^{2}x\left(n-1\right)=f\left(n,x\left(n\right),\Delta x\left(n-1\right)\right),\;n\in N\\ x_{0}-a\Delta x_{0}=B,\;\Delta x\left(\infty \right)=C, \end{array}\right.$$

with a>0, B,C∈R, using an upper and lower solutions method combined with the fixed-point approach and the degree theory. The method of upper and lower solutions on finite intervals with the degree theory was used by Tian et al. in [21] to prove the existence of three solutions to

$$\left\{\begin{array}{c} \Delta^{2}x\left(n-1\right)-p\Delta x\left(n-1\right)-qx\left(n-1\right)+f\left(n,x\left(n\right),\Delta x\left(n\right)\right)=0,\;n\in N\\ x(0)-a\Delta x(l)=B,\;x(n)\;\mathrm{is}\mathrm{bounded}\mathrm{on}\;(0,\infty ), \end{array}\right.$$

with l∈N, B∈R, p≥q>0. For α,β≥0, $\alpha^{2}+\beta^{2}>0$, p,q>0, 1+p>q, Tian and Ge in [20] searched for positive solutions to the following problem:

$$\left\{\begin{array}{c} \Delta^{2}x\left(n-1\right)-p\Delta x\left(n-1\right)-qx\left(n-1\right)+f\left(n,x\left(n\right)\right)=0,\;n\in N\\ \alpha x(0)-\beta \Delta x(0)=0,\;x(\infty )=0 \end{array}\right.$$

via the fixed-point approach in a Fréchet space.

To obtain the main results of this paper we need some auxiliary tools. Let us remind the reader of some of them.

**Theorem** **1** ([22], p. 56)**.** *Let M be a nonempty, closed, bounded, convex subset of a Banach space, and suppose T:M→M is a compact operator. Then, T has a fixed point.*

By $c_{0}$ we denote the Banach space of all sequences convergent to zero, whereas by *c* we denote the Banach space of all convergent sequences. We consider the supremum norm in both spaces.

**Proposition** **1** ([23], p. 107)**.** *A set $A\subset c_{0}$ is relatively compact (with respect to norm topology) if and only if there is a sequence $\left\{\lambda \left(n\right)\right\}\in c_{0}$ such that |x(n)|≤λ(n) for any {x(n)}∈A and for any n∈N∪{0}.*

From Lemma 3.1 in [24] or Lemma 5 in [25] we have:

**Lemma** **1.** *If $\sum_{j=0}^{\infty }\left|g_{j}\right|<\infty$, and one of series $\sum_{k=0}^{\infty }|w_{k}|\sum_{j=k}^{\infty }\left|g_{j}\right|$, $\sum_{k=0}^{\infty }|g_{k}|\sum_{j=0}^{k}\left|w_{j}\right|$ is convergent, then the second series is convergent and*

$$\sum_{k=n}^{\infty }w_{k}\sum_{j=k}^{\infty }g_{j}=\sum_{k=n}^{\infty }g_{k}\sum_{j=n}^{k}w_{j},\;n\in N\cup \left\{0\right\}.$$

The plan of the paper is as follows: Section 2 is devoted to the study of Problem (2) with $\beta \neq \alpha \sum_{l=0}^{\infty }\frac{1}{a(l)}$, and in Section 3 the resonant case is presented.

## 2. Problem without Resonance

This section begins with the presentation of sufficient conditions for the existence of a solution to (2) with $\beta \neq \alpha \sum_{l=0}^{\infty }\frac{1}{a(l)}$ for all d∈R. Assumptions of this case allow us to look for a solution to (2) via a fixed point of an operator defined on some subset of *c*.

**Theorem** **2.** *Let d∈R. Assume that:*

$\left(H_{0}\right)$ *$\beta \neq \alpha \sum_{l=0}^{\infty }\frac{1}{a(l)}$;*
$\left(H_{1}\right)$ *a(l)>0, l∈N∪{0};*
$\left(H_{2}\right)$ *$\sum_{l=0}^{\infty }\frac{l}{a(l)}<\infty$;*
$\left(H_{3}\right)$ *f:N×R×R→R is a continuous function;*
$\left(H_{4}\right)$ *there exists $M>\frac{\left|d\alpha \right|\sum_{l=0}^{\infty }\frac{1}{a(l)}}{\left|\alpha \sum_{l=0}^{\infty }\frac{1}{a(l)}-\beta \right|}$ such that for any n∈N∪{0}*
  $$\max_{|x-d|\leq M,|y|\leq 2M}\left|f\left(n+1,x,y\right)\right|\leq \frac{M-\frac{\left|d\alpha \right|\sum_{l=0}^{\infty }\frac{1}{a(l)}}{\left|\alpha \sum_{l=0}^{\infty }\frac{1}{a(l)}-\beta \right|}}{\left(\sum_{l=0}^{\infty }\frac{l}{a(l)}\right)\left[1+\frac{\left|\alpha \right|\sum_{l=0}^{\infty }\frac{1}{a(l)}}{\left|\alpha \sum_{l=0}^{\infty }\frac{1}{a(l)}-\beta \right|}\right]}.$$

*Then, problem* (2) *possesses a solution.*

**Proof.**  Let *M* satisfy assumption $\left(H_{4}\right)$. By ${\bar{B}}_{M}\left(d\right):=\bar{B}\left(d,M\right)$, we denote the closed ball in *c* with the origin d=(d,d,d,…). In this proof for ${\left\{x\left(n\right)\right\}}_{n\in N\cup \{0\}}\in c$ we use the notation $x:={\left\{x\left(n\right)\right\}}_{n\in N\cup \{0\}}$.

We define an operator $T:{\bar{B}}_{M}\left(d\right)\to c$ as follows:

(3) $$\begin{array}{cc} \; & T\left(x\right)\left(n\right)=d-\sum_{l=n}^{\infty }\frac{1}{a(l)}\sum_{i=0}^{l-1}f\left(i+1,x\left(i+1\right),\Delta x\left(i+1\right)\right)\\ \; & -\frac{\alpha }{\alpha \sum_{l=0}^{\infty }\frac{1}{a(l)}-\beta }\left(\sum_{l=n}^{\infty }\frac{1}{a(l)}\right)\left[d-\sum_{k=0}^{\infty }\frac{1}{a(k)}\sum_{t=0}^{k-1}f\left(t+1,x\left(t+1\right),\Delta x\left(t+1\right)\right)\right] \end{array}$$

for $x={\left\{x\left(n\right)\right\}}_{n\in N\cup \{0\}}\in {\bar{B}}_{M}\left(d\right)$ and n∈N∪{0}. First, we prove under assumptions $\left(H_{2}\right)$ and $\left(H_{4}\right)$ that the operator *T* is well defined. Put

$$F_{n}:=\max_{|x-d|\leq M,|y|\leq 2M}\left|f\left(n+1,x,y\right)\right|,\;n\in N\cup \left\{0\right\};$$

$$K:=\frac{M-\frac{\left|d\alpha \right|\sum_{l=0}^{\infty }\frac{1}{a(l)}}{\left|\alpha \sum_{l=0}^{\infty }\frac{1}{a(l)}-\beta \right|}}{\left(\sum_{l=0}^{\infty }\frac{l}{a(l)}\right)\left[1+\frac{\left|\alpha \right|\sum_{l=0}^{\infty }\frac{1}{a(l)}}{\left|\alpha \sum_{l=0}^{\infty }\frac{1}{a(l)}-\beta \right|}\right]}.$$

Let $x\in {\bar{B}}_{M}\left(d\right)$ and n∈N∪{0}. Hence,

|x(n)−d|≤M,|Δx(n)|≤2M

and

(4) $$\left|\sum_{l=0}^{\infty }\frac{1}{a(l)}\sum_{i=0}^{l-1}f\left(i+1,x\left(i+1\right),\Delta x\left(i+1\right)\right)\right|\leq \sum_{l=0}^{\infty }\frac{1}{a(l)}\sum_{i=0}^{l-1}F_{i}\leq K\sum_{l=0}^{\infty }\frac{l}{a(l)}.$$

The above estimation yields Tx∈c. Now, we show that every fixed point of *T* is a solution to (2). Indeed, let $x\in {\bar{B}}_{M}\left(d\right)$ be a fixed point of *T*; then:

(5) $$\begin{array}{cc} \; & x\left(n\right)=T\left(x\right)\left(n\right)=d-\sum_{l=n}^{\infty }\frac{1}{a(l)}\sum_{i=0}^{l-1}f\left(i+1,x\left(i+1\right),\Delta x\left(i+1\right)\right)\\ \; & -\frac{\alpha }{\alpha \sum_{l=0}^{\infty }\frac{1}{a(l)}-\beta }\left(\sum_{l=n}^{\infty }\frac{1}{a(l)}\right)\left[d-\sum_{k=0}^{\infty }\frac{1}{a(k)}\sum_{t=0}^{k-1}f\left(t+1,x\left(t+1\right),\Delta x\left(t+1\right)\right)\right] \end{array}$$

for n∈N∪{0}. Hence,

$$\begin{array}{cc} \; & a\left(n\right)\Delta x\left(n\right)=\sum_{i=0}^{n-1}f\left(i+1,x\left(i+1\right),\Delta x\left(i+1\right)\right)\\ \; & +\frac{\alpha }{\alpha \sum_{l=0}^{\infty }\frac{1}{a(l)}-\beta }\left[d-\sum_{k=0}^{\infty }\frac{1}{a(k)}\sum_{t=0}^{k-1}f\left(t+1,x\left(t+1\right),\Delta x\left(t+1\right)\right)\right] \end{array}$$

for n∈N∪{0}. Eventually, we obtain that

Δ(a(n)Δx(n))=f(n+1,x(n+1),Δx(n+1))n∈N∪{0}.

Moreover,

$$\begin{array}{cc} \; & x\left(0\right)=T\left(x\right)\left(0\right)=\left(d-\sum_{l=0}^{\infty }\frac{1}{a(l)}\sum_{i=0}^{l-1}f\left(i+1,x\left(i+1\right),\Delta x\left(i+1\right)\right)\right)\left[1-\frac{\alpha \sum_{l=0}^{\infty }\frac{1}{a(l)}}{\alpha \sum_{l=0}^{\infty }\frac{1}{a(l)}-\beta }\right]\\ \; & =\frac{-\beta }{\alpha \sum_{l=0}^{\infty }\frac{1}{a(l)}-\beta }\left(d-\sum_{l=0}^{\infty }\frac{1}{a(l)}\sum_{i=0}^{l-1}f\left(i+1,x\left(i+1\right),\Delta x\left(i+1\right)\right)\right) \end{array}$$

$$a\left(0\right)\Delta x\left(0\right)=\frac{\alpha }{\alpha \sum_{l=0}^{\infty }\frac{1}{a(l)}-\beta }\left(d-\sum_{l=0}^{\infty }\frac{1}{a(l)}\sum_{i=0}^{l-1}f\left(i+1,x\left(i+1\right),\Delta x\left(i+1\right)\right)\right).$$

Hence,

αx(0)+βa(0)Δx(0)=0.

Finally, passing to n→∞ in (5) we obtain $x\left(\infty \right)=\lim_{n\to \infty }x\left(n\right)=d$, which ends the proof that every fixed point of *T* is a solution to (2).

Now, we are in a position to check the assumptions of Schauder’s theorem. We show that $T\left({\bar{B}}_{M}\left(d\right)\right)\subset {\bar{B}}_{M}\left(d\right)$, *T* is continuous and $T({\bar{B}}_{M}\left(d\right))$ is a relatively compact subset of *c*.

Let $x\in {\bar{B}}_{M}\left(d\right)$ and n∈N∪{0}. By the definition of *T* and (4), we have:

$$\begin{array}{cc} \; & \left|T\left(x\right)\left(n\right)-d\right|\leq \sum_{l=0}^{\infty }\frac{1}{a(l)}\sum_{i=0}^{l-1}F_{i}\left[1+\frac{\left|\alpha \right|\sum_{l=0}^{\infty }\frac{1}{a(l)}}{\left|\alpha \sum_{l=0}^{\infty }\frac{1}{a(l)}-\beta \right|}\right]+\frac{\left|\alpha d\right|\sum_{l=0}^{\infty }\frac{1}{a(l)}}{|\alpha \sum_{l=0}^{\infty }\frac{1}{a(l)}-\beta |}\\ \; & \leq K\left(\sum_{l=0}^{\infty }\frac{l}{a(l)}\right)\left[1+\frac{\left|\alpha \right|\sum_{l=0}^{\infty }\frac{1}{a(l)}}{\left|\alpha \sum_{l=0}^{\infty }\frac{1}{a(l)}-\beta \right|}\right]+\frac{\left|\alpha d\right|\sum_{l=0}^{\infty }\frac{1}{a(l)}}{|\alpha \sum_{l=0}^{\infty }\frac{1}{a(l)}-\beta |}=M \end{array}$$

and

$$\left|T\left(x\right)\left(n\right)-d\right|\leq K\sum_{l=n}^{\infty }\frac{l}{a(l)}+\left(\sum_{l=n}^{\infty }\frac{1}{a(l)}\right)\frac{\left|\alpha \right|}{|\alpha \sum_{l=0}^{\infty }\frac{1}{a(l)}-\beta |}\left[d+K\sum_{l=0}^{\infty }\frac{l}{a(l)}\right].$$

From the above we obtain that $T\left({\bar{B}}_{M}\left(d\right)\right)\subset {\bar{B}}_{M}\left(d\right)$ and $\left(T-d\right)({\bar{B}}_{M}\left(d\right))$ is a relatively compact subset of $c_{0}$; see Proposition 1. To prove that *T* is a compact operator in *c*, we have to prove its continuity. Let ε>0. From assumption $\left(H_{2}\right)$ we obtain the existence of $n_{0}\in N$ such that

(6) $$\sum_{l=n_{0}}^{\infty }\frac{l}{a(l)}\leq \frac{\varepsilon }{4K}{\left[1+\frac{\left|\alpha \right|\sum_{l=0}^{\infty }\frac{1}{a(l)}}{\left|\alpha \sum_{l=0}^{\infty }\frac{1}{a(l)}-\beta \right|}\right]}^{-1}.$$

Moreover, there exists η>0 such that

(7) $$\eta \cdot \left(\sum_{l=0}^{n_{0}-1}\frac{l}{a(l)}\right)\leq \frac{\varepsilon }{2}\cdot {\left[1+\frac{\left|\alpha \right|\sum_{l=0}^{\infty }\frac{1}{a(l)}}{\left|\alpha \sum_{l=0}^{\infty }\frac{1}{a(l)}-\beta \right|}\right]}^{-1}.$$

From the uniform continuity of function *f* on $\left\{1,\ldots ,n_{0}-1\right\}\times \left[d-M,d+M\right]\times \left[-2M,2M\right]$ we obtain the existence of δ>0 such that for any $n\in \{0,1,\ldots ,n_{0}-2\}$, $\left(x_{1},y_{1}\right)$, $\left(x_{2},y_{2}\right)$∈[d−M,d+M]×[−2M,2M] and $\left|\right|\left(x_{1},y_{1}\right)-\left(x_{2},y_{2}\right){\left|\right|}_{R^{2}}<3\delta$ (we use the Euclidean norm in $R^{2}$) we have:

$$|f\left(n+1,x_{1},y_{1}\right)-f\left(n+1,x_{2},y_{2}\right)|<\eta .$$

Let $x,z\in {\bar{B}}_{M}\left(d\right)$, ||x−z||<δ and n∈N∪{0}. Notice that for any n∈N∪{0} we obtain:

|x(n)−z(n)|<δ,|Δx(n)−Δz(n)|<2δ

and

$$\begin{array}{cc} \; & \left|T(x)(n)-T(z)(n)\right|\leq \left[1+\frac{\left|\alpha \right|\sum_{l=0}^{\infty }\frac{1}{a(l)}}{\left|\alpha \sum_{l=0}^{\infty }\frac{1}{a(l)}-\beta \right|}\right]\cdot \\ \; & \sum_{l=0}^{\infty }\frac{1}{a(l)}\sum_{i=0}^{l-1}\left|f(i+1,x(i+1),\Delta x(i+1))-f(i+1,z(i+1),\Delta z(i+1))\right|\\ \; & \leq \left[1+\frac{\left|\alpha \right|\sum_{l=0}^{\infty }\frac{1}{a(l)}}{\left|\alpha \sum_{l=0}^{\infty }\frac{1}{a(l)}-\beta \right|}\right]\cdot \left[\sum_{l=0}^{n_{0}-1}\frac{1}{a(l)}\sum_{i=0}^{l-1}\eta +\sum_{l=n_{0}}^{\infty }\frac{1}{a(l)}\sum_{i=0}^{l-1}2F_{i}\right]\\ \; & \leq \left[1+\frac{\left|\alpha \right|\sum_{l=0}^{\infty }\frac{1}{a(l)}}{\left|\alpha \sum_{l=0}^{\infty }\frac{1}{a(l)}-\beta \right|}\right]\cdot \left[\eta \sum_{l=0}^{n_{0}-1}\frac{l}{a(l)}+2K\sum_{l=n_{0}}^{\infty }\frac{l}{a(l)}\right]. \end{array}$$

From (6) and (7) above, we have

$$||Tx-Tz||=\underset{n\in N\cup \{0\}}{\sup}\left|T(x)(n)-T(z)(n)\right|<\varepsilon .$$

By Schauder’s theorem we obtain that there exists a fixed point $x\in {\bar{B}}_{M}\left(d\right)$ of *T*, which is a solution to (2). □

**Corollary** **1.** *Suppose that the assumptions of Theorem 2 are satisfied with d=0. Moreover, assume that*

$\left(H_{5}\right)$ *$f(n_{0}+1,0,0)\neq 0$ for some $n_{0}\in N\cup \left\{0\right\}$.*

*Then, problem* (2) *possesses a nontrivial solution.*

**Remark** **1.** *Note that $\left(H_{2}\right)$ implies that $\lim_{n\to \infty }a\left(n\right)=\infty$. Moreover, if $\frac{1}{a(n)}=O\left(n^{-2-\varepsilon }\right)$, for ε>0, then $\left(H_{2}\right)$ is satisfied.*

We will now present examples of classes of functions which satisfy $\left(H_{4}\right)$.

**Example** **1.** *Let d∈R and $f:N\times R^{2}\to R$ be a continuous function fulfilling*

(8) |f(n+1,x,y)|≤b(n)|x−d|+c(n)|y|+e(n)forn∈N∪{0},x,y∈R,

*with nonnegative sequences {b(n)}, {c(n)}, {e(n)} such that*

(9) $$\begin{array}{cc} \; & \underset{n\in N\cup \{0\}}{\sup}\left(b(n)+2c(n)\right)<{\left(\sum_{l=0}^{\infty }\frac{l}{a(l)}\right)}^{-1}\cdot {\left[1+\frac{\left|\alpha \right|\sum_{l=0}^{\infty }\frac{1}{a(l)}}{\left|\alpha \sum_{l=0}^{\infty }\frac{1}{a(l)}-\beta \right|}\right]}^{-1}, \end{array}$$

(10) $$\begin{array}{cc} \; & \underset{n\in N\cup \{0\}}{\sup}\frac{\frac{\left|d\alpha \right|\sum_{l=0}^{\infty }\frac{1}{a(l)}}{\left|\alpha \sum_{l=0}^{\infty }\frac{1}{a(l)}-\beta \right|}+e\left(n\right)\left(\sum_{l=0}^{\infty }\frac{l}{a(l)}\right)\left[1+\frac{\left|\alpha \right|\sum_{l=0}^{\infty }\frac{1}{a(l)}}{\left|\alpha \sum_{l=0}^{\infty }\frac{1}{a(l)}-\beta \right|}\right]}{1-\left(\sum_{l=0}^{\infty }\frac{l}{a(l)}\right)\left[1+\frac{\left|\alpha \right|\sum_{l=0}^{\infty }\frac{1}{a(l)}}{\left|\alpha \sum_{l=0}^{\infty }\frac{1}{a(l)}-\beta \right|}\right]\left(b\left(n\right)+2c\left(n\right)\right)}<+\infty . \end{array}$$

*It is easy to see, that for*

(11) $$M\geq \underset{n\in N\cup \{0\}}{\sup}\frac{\frac{\left|d\alpha \right|\sum_{l=0}^{\infty }\frac{1}{a(l)}}{\left|\alpha \sum_{l=0}^{\infty }\frac{1}{a(l)}-\beta \right|}+e\left(n\right)\left(\sum_{l=0}^{\infty }\frac{l}{a(l)}\right)\left[1+\frac{\left|\alpha \right|\sum_{l=0}^{\infty }\frac{1}{a(l)}}{\left|\alpha \sum_{l=0}^{\infty }\frac{1}{a(l)}-\beta \right|}\right]}{1-\left(\sum_{l=0}^{\infty }\frac{l}{a(l)}\right)\left[1+\frac{\left|\alpha \right|\sum_{l=0}^{\infty }\frac{1}{a(l)}}{\left|\alpha \sum_{l=0}^{\infty }\frac{1}{a(l)}-\beta \right|}\right]\left(b\left(n\right)+2c\left(n\right)\right)}$$

*assumption $\left(H_{4}\right)$ of Theorem 2 is satisfied.*

*Note that, for any L>0, condition* (10) *with b(n)=c(n)=0 and e(n)=L, n∈N∪{0} is satisfied. It means that this case includes a class of bounded functions. Moreover, $\left(H_{4}\right)$ holds for a linear function with respect to second and third variables, i.e.,*

f(n+1,x,y)=b(n)(x−d)+c(n)(y)+e(n)forn∈N∪{0},x,y∈R,

*where {b(n)}, {c(n)} satisfy* (9) *and {e(n)} is bounded.*

The next example is a simple consequence of Example 1.

**Example** **2.** *Let d∈R and $f:N\times R^{2}\to R$ be a continuous function with a sublinear growth with respect to second and third variables, i.e.,*

$$\left|f(n+1,x,y)\right|\leq {b\left(n\right)|x-d|}^{\delta }+c\left(n\right){\left|y\right|}^{\gamma }+e\left(n\right)\;\mathrm{for}\;n\in N\cup \left\{0\right\},\;x,y\in R,$$

*with δ,γ∈(0,1], nonnegative sequences {b(n)}, {c(n)}, {e(n)} fulfill* (9), (10). *In this case, assumption $\left(H_{4}\right)$ of Theorem 2 is satisfied with M>1 such that* (11).

Let us remind the reader that in the classical approach we assume that $\sum_{l=0}^{\infty }\frac{1}{a(l)}<\infty$. To see that our condition $\sum_{l=0}^{\infty }\frac{l}{a(l)}<\infty$ is not too strong we present the following necessary condition for the existence of a solution to (2). It is worth mentioning that the following necessary condition is true in both cases when Problem (2) is with and without resonance.

**Theorem** **3.** *Let d∈R. Suppose that Problem* (2) *possesses a solution. Moreover, assume that:*

$\left(H_{1}\right)$ *a(l)>0, l∈N∪{0};*
$\left(H_{6}\right)$ *there exist $n_{0}\in N\cup \left\{0\right\}$, K>0 such that*
  $$\eta :=\min\{f\left(n+1,x,y\right):n\geq n_{0},\;|x-d|\leq K,\;|y|\leq 2K\}>0.$$

*Then,*

$$\sum_{l=0}^{\infty }\frac{l}{a(l)}<\infty .$$

**Proof.**  If ${\left\{x\left(n\right)\right\}}_{n\in N\cup \{0\}}$ is a nontrivial solution to (2), then there exists $n_{1}\in N$ such that |x(n)−d|≤K for any $n\geq n_{1}$. Then |Δx(n)|≤2K for any $n\geq n_{1}$ and summing up from $n_{2}:=\max\left\{n_{1},n_{0}\right\}$ to n−1 from the equation in (2) we obtain:

$$a\left(n\right)\Delta x\left(n\right)-a\left(n_{2}\right)\Delta x\left(n_{2}\right)=\sum_{k=n_{2}}^{n-1}f\left(k+1,x\left(k+1\right),\Delta x\left(k+1\right)\right)$$

for $n\geq n_{2}$. Hence, we have:

$$\Delta x\left(n\right)=\frac{a\left(n_{2}\right)\Delta x\left(n_{2}\right)+\sum_{k=n_{2}}^{n-1}f\left(k+1,x\left(k+1\right),\Delta x\left(k+1\right)\right)}{a(n)}.$$

Summing up the above from $n_{2}$ to n−1 and using $\left(H_{6}\right)$, we obtain:

$$x\left(n\right)-x\left(n_{2}\right)=\sum_{l=n_{2}}^{n}\frac{a\left(n_{2}\right)\Delta x\left(n_{2}\right)+\sum_{k=n_{2}}^{l-1}f\left(k+1,x\left(k+1\right),\Delta x\left(k+1\right)\right)}{a(l)}\geq \sum_{l=n_{2}}^{n}\frac{a\left(n_{2}\right)\Delta x\left(n_{2}\right)+\eta \left(l-n_{2}\right)}{a(l)}$$

for $n>n_{2}$. Using the fact that $x\left(+\infty \right)=\lim_{n\to \infty }x\left(n\right)=d$ and letting n→∞ in the above, we have:

$$d-x\left(n_{2}\right)\geq \sum_{l=n_{2}}^{\infty }\left(\frac{\eta l+a\left(n_{2}\right)\Delta x\left(n_{2}\right)-\eta n_{2}}{a(l)}\right).$$

By the positivity of η there exists $n_{3}\in N$, $n_{3}>n_{2}$ such that

$$\eta l+a\left(n_{2}\right)\Delta x\left(n_{2}\right)-\eta n_{2}>\frac{\eta }{2}l$$

for $l\geq n_{3}$. Hence,

$$d-x\left(n_{2}\right)\geq \sum_{l=n_{2}}^{n_{3}-1}\left(\frac{\eta l+a\left(n_{2}\right)\Delta x\left(n_{2}\right)-\eta n_{2}}{a(l)}\right)+\frac{\eta }{2}\sum_{l=n_{3}}^{\infty }\frac{l}{a(l)}$$

and

$$\sum_{l=0}^{\infty }\frac{l}{a(l)}<\infty .$$

□

## 3. Problem with Resonance

Assuming that $\sum_{l=0}^{\infty }\frac{l}{a(l)}<\infty$, the following problem:

(12) $$\left\{\begin{array}{c} \Delta \left(a(n)\Delta x(n)\right)=f\left(n+1,x\left(n+1\right),\Delta x\left(n+1\right)\right),\;n\in N\cup \left\{0\right\},\\ x\left(0\right)+a\left(0\right)\left(\sum_{l=0}^{\infty }\frac{1}{a(l)}\right)\Delta x\left(0\right)=0,\;x\left(\infty \right)=0 \end{array}\right.$$

can be written in the abstract form Lx=Nx, where $L:c_{0}⊃\mathrm{dom}L\to l_{\infty }\times R$, $l_{\infty }$ is the space of bounded sequences,

$$\left(Lx\right)\left(n\right)=\left(\Delta \left(a(n)\Delta x(n)\right),x\left(0\right)+a\left(0\right)\left(\sum_{l=0}^{\infty }\frac{1}{a(l)}\right)\Delta x\left(0\right)\right),$$

$\mathrm{dom}L=\{\left\{x\left(n\right)\right\}\in c_{0}:\left\{\Delta \left(a\left(n\right)\Delta x\left(n\right)\right)\right\}\in l_{\infty }\}$. It is obvious that $\ker L=\{\left\{x\left(n\right)\right\}\in c_{0}:x\left(n\right)=-d_{1}\sum_{l=n}^{\infty }\frac{1}{a(l)},\;n\in N\cup \left\{0\right\},\;d_{1}\in R\}$ and $\mathrm{im}L=\{\left(\left\{y\left(n\right)\right\},z\right)\in l_{\infty }\times R:z=\sum_{l=0}^{\infty }\frac{1}{a(l)}\sum_{i=0}^{l-1}y\left(i\right),\;\left\{y\left(n\right)\right\}\in l_{\infty }\}$. Hence, dimkerL=1, codimimL=1 and indL:=dimkerL−codimimL=0, where indL denotes an index of an operator *L*. This means that *L* is a Fredholm operator of index 0. To establish sufficient conditions for the existence of a solution to (12) we use the Przeradzki perturbation method with the perturbation of the first boundary condition $x\left(0\right)+a\left(0\right)\left(\sum_{l=0}^{\infty }\frac{1}{a(l)}\right)\Delta x\left(0\right)=0$. This approach allows us to work not only for (12), but by the translation to sequence d=(d,d,d…) in space *c* with the more general problem

(13) $$\left\{\begin{array}{c} \Delta \left(a(n)\Delta x(n)\right)=f\left(n+1,x\left(n+1\right),\Delta x\left(n+1\right)\right),\;n\in N\cup \left\{0\right\},\\ x\left(0\right)+a\left(0\right)\left(\sum_{l=0}^{\infty }\frac{1}{a(l)}\right)\Delta x\left(0\right)=0,\;x\left(\infty \right)=d, \end{array}\right.$$

with d∈R. We obtain the following theorem.

**Theorem** **4.** *Let d∈R. Assume that:*

$\left(H_{1}\right)$ *a(l)>0, l∈N∪{0};*
$\left(H_{2}\right)$ *$\sum_{l=0}^{\infty }\frac{l}{a(l)}<\infty$;*
$\left(H_{3}^{\prime }\right)$ *f:N×R×R→R is a continuous and bounded function;*
$\left(H_{7}\right)$ *there exists M>0 such that u(f(n+1,u,v)−d)>0 for all n∈N∪{0}, |u|≥M, v∈R;*
$\left(H_{8}\right)$ *there exists $n_{0}\in N\cup \left\{0\right\}$ such that $\lim_{|u|\to \infty }f\left(n_{0}+1,u,v\right)\neq d$ for all v∈R.*

*Then, problem* (13) *possesses a solution.*

**Proof.**  By Lemma 1, $\left(H_{2}\right)$ implies that $\sum_{l=0}^{\infty }\sum_{i=l+1}^{\infty }\frac{1}{a(i)}=\sum_{l=0}^{\infty }\frac{l}{a(l)}<\infty$. Dividing the equation and the first boundary condition in (13) by $\sum_{l=0}^{\infty }\sum_{i=l+1}^{\infty }\frac{1}{a(i)}$, if necessary, we assume that

(14) $$\sum_{l=0}^{\infty }\sum_{i=l+1}^{\infty }\frac{1}{a(i)}=1.$$

Let k∈N. We consider the perturbed problem

(15) $$\left\{\begin{array}{c} \Delta \left(a(n)\Delta x(n)\right)=f\left(n+1,x\left(n+1\right),\Delta x\left(n+1\right)\right),\;n\in N\cup \left\{0\right\},\\ \left(1-\frac{1}{k}\right)x\left(0\right)+a\left(0\right)\left(\sum_{l=0}^{\infty }\frac{1}{a(l)}\right)\Delta x\left(0\right)=0,\;x\left(\infty \right)=d \end{array}\right.$$

under (14). Notice that after dividing (15) by $\sum_{l=0}^{\infty }\sum_{i=l+1}^{\infty }\frac{1}{a(i)}$, the nonlinear function *f* is still bounded. Hence, there exists L>0 such that |f(n,x,y)|≤L for n∈N∪{0}, x,y∈R. It is clear that problem (15) satisfies assumptions of Theorem 2 with $M_{k}:=kL\left(\sum_{l=0}^{\infty }\frac{l}{a(l)}\right)+|d|\left(k-1\right)=kL+|d|\left(k-1\right)$. Hence, there exists a solution $x^{k}$ to (15). We prove that $\left\{|\right|x^{k}{\left||\right\}}_{k\in N}$ is bounded in *c*. On the contrary, suppose that $\left\{|\right|x^{k}{\left||\right\}}_{k\in N}$ is unbounded. Passing to subsequence if necessary we assume that $\left|\right|x^{k}\left|\right|\to \infty$, as k→∞. Dividing (15) by $\left|\right|x^{k}\left|\right|$ we obtain:

(16) $$\left\{\begin{array}{c} \Delta \left(a\left(n\right)\Delta \frac{x^{k}\left(n\right)}{\left|\right|x^{k}\left|\right|}\right)=\frac{f(n+1,x^{k}\left(n+1\right),\Delta x^{k}\left(n+1\right))}{\left|\right|x^{k}\left|\right|},\;n\in N\cup \left\{0\right\},\\ \left(1-\frac{1}{k}\right)\frac{x^{k}\left(0\right)}{\left|\right|x^{k}\left|\right|}+a\left(0\right)\left(\sum_{l=0}^{\infty }\frac{1}{a(l)}\right)\Delta \frac{x^{k}\left(0\right)}{\left|\right|x^{k}\left|\right|}=0,\;x^{k}\left(\infty \right)=d \end{array}\right.$$

for any k∈N. By the boundedness of *f* there exists $M_{1}>0$ such that

$$-M_{1}\leq \Delta \left(a\left(n\right)\Delta \frac{x^{k}\left(n\right)}{\left|\right|x^{k}\left|\right|}\right)\leq M_{1}$$

for any n∈N∪{0}, k∈N. Summing the above from 0 to n−1 we have

$$-M_{1}n\leq a\left(n\right)\Delta \frac{x^{k}\left(n\right)}{\left|\right|x^{k}\left|\right|}-a\left(0\right)\Delta \frac{x^{k}\left(0\right)}{\left|\right|x^{k}\left|\right|}\leq M_{1}n$$

and

$$-M_{1}n-2a\left(0\right)\leq a\left(n\right)\Delta \frac{x^{k}\left(n\right)}{\left|\right|x^{k}\left|\right|}\leq M_{1}n+2a\left(0\right)$$

for any n∈N∪{0}, k∈N. Then,

$$\frac{-M_{1}n-2a\left(0\right)}{a(n)}\leq \Delta \frac{x^{k}\left(n\right)}{\left|\right|x^{k}\left|\right|}\leq \frac{M_{1}n+2a\left(0\right)}{a(n)}$$

for any n∈N∪{0}, k∈N. Summing the above from *n* to m−1, (m−1>n) we obtain:

$$\sum_{l=n}^{m-1}\frac{-M_{1}l-2a\left(0\right)}{a(l)}\leq \frac{x^{k}\left(m\right)-x^{k}\left(n\right)}{\left|\right|x^{k}\left|\right|}\leq \sum_{l=n}^{m-1}\frac{M_{1}l+2a\left(0\right)}{a(l)}$$

for any n∈N∪{0}, k∈N. Passing to m→∞ we obtain:

$$\sum_{l=n}^{\infty }\frac{-M_{1}l-2a\left(0\right)}{a(l)}\leq \frac{d-x^{k}\left(n\right)}{\left|\right|x^{k}\left|\right|}\leq \sum_{l=n}^{\infty }\frac{M_{1}l+2a\left(0\right)}{a(l)}$$

for any n∈N∪{0}, k∈N. This means that ${\left\{\frac{x^{k}-d}{\left|\right|x^{k}\left|\right|}\right\}}_{k\in N}$ is a relatively compact sequence in $c_{0}$, where d=(d,d,d,…)∈c; see Proposition 1. Passing to subsequence if necessary we assume that there exists $x^{0}\in c_{0}$ with $\left|\right|x^{0}\left|\right|=1$ such that $\lim_{k\to \infty }\frac{x^{k}-d}{\left|\right|x^{k}\left|\right|}=x^{0}$ in $c_{0}$. Taking into account that $\left|\right|x^{k}\left|\right|\to \infty$ and the above we have $\lim_{k\to \infty }\frac{x^{k}}{\left|\right|x^{k}\left|\right|}=x^{0}$ in *c*. Passing to k→∞ in (16) we obtain that

$$\left\{\begin{array}{c} \Delta \left(a\left(n\right)\Delta x^{0}\left(n\right)\right)=0,\;n\in N\cup \left\{0\right\}\\ x^{0}\left(0\right)+a\left(0\right)\left(\sum_{l=0}^{\infty }\frac{1}{a(l)}\right)\Delta x^{0}\left(0\right)=0. \end{array}\right.$$

It is easy to see that

$$\left(x^{0}\left(n\right)=\frac{\sum_{l=n}^{\infty }\frac{1}{a(l)}}{\sum_{l=0}^{\infty }\frac{1}{a(l)}},\;n\in N\cup \left\{0\right\}\right)\;\vee \;\left(x^{0}\left(n\right)=-\frac{\sum_{l=n}^{\infty }\frac{1}{a(l)}}{\sum_{l=0}^{\infty }\frac{1}{a(l)}},\;n\in N\cup \left\{0\right\}\right).$$

Let us assume that $x^{0}\left(n\right)=\frac{\sum_{l=n}^{\infty }\frac{1}{a(l)}}{\sum_{l=0}^{\infty }\frac{1}{a(l)}}$, n∈N∪{0}. Knowing that $x^{k}$, k∈N is a fixed point of operator (3), we obtain from Theorem 4 that

(17) $$\begin{array}{cc} \; & \frac{x^{k}\left(n\right)}{\left|\right|x^{k}\left|\right|}=\frac{d}{\left|\right|x^{k}\left|\right|}-\frac{1}{\left|\right|x^{k}\left|\right|}\sum_{l=n}^{\infty }\frac{1}{a(l)}\sum_{i=0}^{l-1}f\left(i+1,x^{k}\left(i+1\right),\Delta x^{k}\left(i+1\right)\right)\\ \; & +\frac{k-1}{\left|\right|x^{k}\left|\right|}x^{0}\left(n\right)\left[d-\sum_{t=0}^{\infty }\frac{1}{a(t)}\sum_{s=0}^{t-1}f\left(s+1,x^{k}\left(s+1\right),\Delta x^{k}\left(s+1\right)\right)\right] \end{array}$$

for any n∈N∪{0}, k∈N. By the boundedness of *f* and $\left(H_{2}\right)$, Lemma 1 and (14) we have:

(18) $$\begin{array}{cc} \; & 1=\lim_{k\to \infty }\frac{k-1}{\left|\right|x^{k}\left|\right|}\left(d-\sum_{t=0}^{\infty }\frac{1}{a(t)}\sum_{s=0}^{t-1}f\left(s+1,x^{k}\left(s+1\right),\Delta x^{k}\left(s+1\right)\right)\right)\\ \; & =\lim_{k\to \infty }\frac{k-1}{\left|\right|x^{k}\left|\right|}\left(d-\sum_{t=0}^{\infty }f\left(t+1,x^{k}\left(t+1\right),\Delta x^{k}\left(t+1\right)\right)\sum_{s=t+1}^{\infty }\frac{1}{a(s)}\right)\\ \; & =\lim_{k\to \infty }\frac{k-1}{\left|\right|x^{k}\left|\right|}\sum_{t=0}^{\infty }\left[\left(d-f(t+1,x^{k}\left(t+1\right),\Delta x^{k}\left(t+1\right))\right)\sum_{s=t+1}^{\infty }\frac{1}{a(s)}\right]. \end{array}$$

Hence, there exists $k_{0}$ such that

(19) $$\sum_{t=0}^{\infty }\left[\left(d-f(t+1,x^{k}\left(t+1\right),\Delta x^{k}\left(t+1\right))\right)\sum_{s=t+1}^{\infty }\frac{1}{a(s)}\right]>0$$

for any $k\geq k_{0}$. Using Fatou’s lemma with summable lower bound ${\left\{\left(-L-d\right)\sum_{s=n+1}^{\infty }\frac{1}{a(s)}\right\}}_{n\in N\cup \{0\}}$, we obtain that

$$\begin{array}{cc} \; & \sum_{t=0}^{\infty }\underset{k\to \infty }{lim inf}\left[\left(f(t+1,x^{k}\left(t+1\right),\Delta x^{k}\left(t+1\right))-d\right)\sum_{s=t+1}^{\infty }\frac{1}{a(s)}\right]\\ \; & \leq \underset{k\to \infty }{lim inf}\sum_{t=0}^{\infty }\left[\left(f(t+1,x^{k}\left(t+1\right),\Delta x^{k}\left(t+1\right))-d\right)\sum_{s=t+1}^{\infty }\frac{1}{a(s)}\right]\leq 0. \end{array}$$

We consider two cases.

Case 1. $\sum_{t=0}^{\infty }{lim inf}_{k\to \infty }\left[\left(f(t+1,x^{k}\left(t+1\right),\Delta x^{k}\left(t+1\right))-d\right)\sum_{s=t+1}^{\infty }\frac{1}{a(s)}\right]<0$. There exists $t_{0}\in N\cup \left\{0\right\}$ such that

$$\underset{k\to \infty }{lim inf}\left(f(t_{0}+1,x^{k}\left(t_{0}+1\right),\Delta x^{k}\left(t_{0}+1\right))-d\right)\sum_{s=t_{0}+1}^{\infty }\frac{1}{a(s)}<0.$$

Passing to subsequence, if necessary we obtain that

(20) $$\lim_{k\to \infty }f\left(t_{0}+1,x^{k}\left(t_{0}+1\right),\Delta x^{k}\left(t_{0}+1\right)\right)<d.$$

Taking into account that $\lim_{k\to \infty }\frac{x^{k}}{\left|\right|x^{k}\left|\right|}=x^{0}$ in *c*, we have that $\lim_{k\to \infty }\frac{x^{k}\left(t_{0}+1\right)}{\left|\right|x^{k}\left|\right|}=x^{0}\left(t_{0}+1\right)>0$. By $\left|\right|x^{k}\left|\right|\to \infty$ and the above there exists $\hat{k}\in N$ such that $\frac{1}{2}x^{0}\left(t_{0}+1\right)\left|\right|x^{k}\left|\right|>M$ and

$$x^{k}\left(t_{0}+1\right)\geq \frac{1}{2}x^{0}\left(t_{0}+1\right)\left|\right|x^{k}\left|\right|>M$$

for $k\geq \hat{k}$. By $\left(H_{7}\right)$ we obtain that

$$f(t_{0}+1,x^{k}\left(t_{0}+1\right),\Delta x^{k}\left(t_{0}+1\right))>d$$

for $k\geq \hat{k}$, which contradicts (20). This excludes Case 1.

Case 2. $\sum_{t=0}^{\infty }{lim inf}_{k\to \infty }\left[\left(f(t+1,x^{k}\left(t+1\right),\Delta x^{k}\left(t+1\right))-d\right)\sum_{s=t+1}^{\infty }\frac{1}{a(s)}\right]=0$. There exist $t_{1},t_{2}\in N\cup \left\{0\right\}$ such that -4.6cm0cm

(21) $$\underset{k\to \infty }{lim inf}f\left(t_{1}+1,x^{k}\left(t_{1}+1\right),\Delta x^{k}\left(t_{1}+1\right)\right)<d\;\wedge \;\underset{k\to \infty }{lim inf}f\left(t_{2}+1,x^{k}\left(t_{2}+1\right),\Delta x^{k}\left(t_{2}+1\right)\right)>d$$

or

(22) $$\underset{k\to \infty }{lim inf}f\left(t+1,x^{k}\left(t+1\right),\Delta x^{k}\left(t+1\right)\right)=d,\;t\in N\cup \left\{0\right\}.$$

We exclude (21) in the same way as in Case 1. On the other hand, for $t=n_{0}$, we have that

$$x^{k}\left(n_{0}+1\right)\geq \frac{1}{2}x^{0}\left(n_{0}+1\right)\left|\right|x^{k}\left|\right|$$

for all large *k*. Hence, we exclude (22) by $\left(H_{8}\right)$.

This means that $x^{0}\left(n\right)=\frac{\sum_{l=n}^{\infty }\frac{1}{a(l)}}{\sum_{l=0}^{\infty }\frac{1}{a(l)}}$, n∈N∪{0} is impossible. For $x^{0}\left(n\right)=-\frac{\sum_{l=n}^{\infty }\frac{1}{a(l)}}{\sum_{l=0}^{\infty }\frac{1}{a(l)}}$, n∈N∪{0} we obtain that $x^{k}$, k∈N is a fixed point of operator (3) from Theorem 2, and hence

(23) $$\begin{array}{cc} \; & \frac{x^{k}\left(n\right)}{\left|\right|x^{k}\left|\right|}=\frac{d}{\left|\right|x^{k}\left|\right|}-\frac{1}{\left|\right|x^{k}\left|\right|}\sum_{l=n}^{\infty }\frac{1}{a(l)}\sum_{i=0}^{l-1}f\left(i+1,x^{k}\left(i+1\right),\Delta x^{k}\left(i+1\right)\right)\\ \; & -\frac{k-1}{\left|\right|x^{k}\left|\right|}x^{0}\left(n\right)\left[d-\sum_{t=0}^{\infty }\frac{1}{a(t)}\sum_{s=0}^{t-1}f\left(s+1,x^{k}\left(s+1\right),\Delta x^{k}\left(s+1\right)\right)\right] \end{array}$$

for any n∈N∪{0}, k∈N. Hence, we obtain

(24) $$\begin{array}{cc} \; & -1=\lim_{k\to \infty }\frac{k-1}{\left|\right|x^{k}\left|\right|}\left(d-\sum_{t=0}^{\infty }\frac{1}{a(t)}\sum_{s=0}^{t-1}f\left(s+1,x^{k}\left(s+1\right),\Delta x^{k}\left(s+1\right)\right)\right)\\ \; & =\lim_{k\to \infty }\frac{k-1}{\left|\right|x^{k}\left|\right|}\left(d-\sum_{t=0}^{\infty }f\left(t+1,x^{k}\left(t+1\right),\Delta x^{k}\left(t+1\right)\right)\sum_{s=t+1}^{\infty }\frac{1}{a(s)}\right)\\ \; & =\lim_{k\to \infty }\frac{k-1}{\left|\right|x^{k}\left|\right|}\sum_{t=0}^{\infty }\left[\left(d-f(t+1,x^{k}\left(t+1\right),\Delta x^{k}\left(t+1\right))\right)\sum_{s=t+1}^{\infty }\frac{1}{a(s)}\right]. \end{array}$$

Similarly to $x^{0}\left(n\right)=\frac{\sum_{l=n}^{\infty }\frac{1}{a(l)}}{\sum_{l=0}^{\infty }\frac{1}{a(l)}}$, n∈N∪{0} we exclude $x^{0}\left(n\right)=-\frac{\sum_{l=n}^{\infty }\frac{1}{a(l)}}{\sum_{l=0}^{\infty }\frac{1}{a(l)}}$, n∈N∪{0}.

This contradiction means that ${\left\{x^{k}\right\}}_{k\in N}$ is a bounded sequence in *c*. Hence, there exists $M_{2}>0$ such that

(25) $$-L\leq \Delta \left(a\left(n\right)\Delta x^{k}\left(n\right)\right)\leq L,\;||x^{k}\left|\right|\leq M_{2}$$

for any n∈N∪{0}, k∈N. Summing the above from 0 to n−1 we obtain:

$$-Ln\leq a\left(n\right)\Delta x^{k}\left(n\right)-a\left(0\right)\Delta x^{k}\left(0\right)\leq Ln$$

and

$$-Ln-2M_{2}a\left(0\right)\leq a\left(n\right)\Delta x^{k}\left(n\right)\leq Ln+2a\left(0\right)M_{2}$$

for any n∈N∪{0}, k∈N. Then,

$$\frac{-Ln-2M_{2}a\left(0\right)}{a(n)}\leq \Delta x^{k}\left(n\right)\leq \frac{Ln+2M_{2}a\left(0\right)}{a(n)}$$

for any n∈N∪{0}, k∈N. Summing the above from *n* to *∞* we obtain

$$\sum_{l=n}^{\infty }\frac{-Ll-2M_{2}a\left(0\right)}{a(l)}\leq d-x^{k}\left(n\right)\leq \sum_{l=n}^{\infty }\frac{Ll+2M_{2}a\left(0\right)}{a(l)}$$

for any n∈N∪{0}, k∈N. This means that ${\left\{x^{k}-d\right\}}_{k\in N}$ is a relatively compact sequence in $c_{0}$. Passing to subsequence if necessary we assume that there exists $\hat{x}\in c_{0}$ such that $\lim_{k\to \infty }x^{k}=\hat{x}+d$ in *c*. Let us denote $\bar{x}:=\hat{x}+d$. By the continuity of *f* and the fact that $\bar{x}\left(\infty \right)=d$, passing to k→∞ in (16) we have that

$$\left\{\begin{array}{c} \Delta \left(a\left(n\right)\Delta \bar{x}\left(n\right)\right)=f\left(n+1,\bar{x}\left(n+1\right),\Delta \bar{x}\left(n+1\right)\right),\;n\in N\cup \left\{0\right\}\\ \bar{x}\left(0\right)+a\left(0\right)\left(\sum_{l=0}^{\infty }\frac{1}{a(l)}\right)\Delta \bar{x}\left(0\right)=0,\;\bar{x}\left(\infty \right)=d, \end{array}\right.$$

which means that $\bar{x}$ is a solution to (13). □

**Example** **3.** *Let d∈R. Note that a continuous function $f:N\times R^{2}\to R$ such that*

$$f\left(n+1,x,y\right)=\arctan\left(x\left(n^{2}+y^{2}+1\right)\right)+d,\;n\in N\cup \left\{0\right\},\;y\in R,\;\left|x\right|\geq 1$$

*satisfies assumptions $\left(H_{3}^{\prime }\right)$, $\left(H_{7}\right)$, and $\left(H_{8}\right)$ of Theorem 4.*

## 4. Conclusions

In this paper, we constructed sufficient conditions for the existence of a solution to the discrete boundary value problem on the half-line (2) in dependence on parameters α,β,d∈R. For α=0, d∈(0,1) the considered problem can be interpreted as a discrete version of some problem from hydrodynamics; see [2]. The fixed-point approach is used when problem (2) is without resonance. In the resonant case the considered problem can be solved via the perturbation technique for a Fredholm operator of index 0. We proved that the constructed assumptions are not too strong by providing the necessary condition for the existence of a solution to this problem.

## Data Availability

Not applicable.

## References

[1] Agarwal R.P., O’Regan D. (2001). Infinite Interval Problems for Differential, Difference and Integral Equations.

[2] Rachůnek L., Rachůnková I. (2011). Homoclinic solutions of non-autonomous difference equations arising in hydrodynamics. Nonlinear Anal..

[3] Bachar I., Mâagli H. (2014). Existence and global asymptotic behavior of positive solutions for nonlinear problems on the half-line. J. Math. Anal. Appl..

[4] Guseinov G.S., Yaslan I. (2004). Boundary value problems for second order nonlinear differential equations on infinite intervals. J. Math. Anal. Appl..

[5] Lian H., Ge W. (2006). Existence of positive solutions for Sturm-Liouville boundary value problems on the half-line. J. Math. Anal. Appl..

[6] Sun Y., Sun Y., Debnath L. (2009). On the existence of positive solutions for singular boundary value problems on the half-line. Appl. Math. Lett..

[7] Yan B., Liu Y. (2004). Unbounded solutions of the singular boundary value problems for second order differential equations on the half-line. Appl. Math. Comput..

[8] Bachar I. (2015). Combined effects in nonlinear singular second order differential equations problems on the half-line. Electron. J. Differ. Equ..

[9] Djebali S., Mebarki K. (2008). Multiple positive solutions for singular BVPs on the positive half-line. Comput. Math. Appl..

[10] Moussaoui T., Szymańska-Dȩbowska K. (2009). Resonant problem for a class of BVPs on the half-line. Electron J. Qual. Theory Diff. Equ..

[11] Szymańska-Dȩbowska K. (2007). Resonant problem for some second-order differential equation on the half-line. Electron. J. Differ. Equ..

[12] Przeradzki B. (1993). Three methods for the study of semilinear equations at resonance. Coll. Math..

[13] Przeradzki B. (1993). A new continuation method for the study of nonlinear equations at resonance. J. Math. Anal. Appl..

[14] Mawhin J. (1979). Topological Degree Methods in Nonlinear Boundary Value Problems.

[15] Berkani M., Castro-González N. (2008). Unbounded B-Fredholm operator on Hilbert spaces. Proc. Edinburgh Math. Soc..

[16] Hammad H.A., De la Sen M. (2019). A solution of Fredholm integral equation by using the cyclic $\eta_{s}^{q}$-rational contractive mappings technique in *b*-metric-like spaces. Symmetry.

[17] Došlá Z., Marini M., Matucci S. (2016). Decaying solutions for discrete boundary value problems on the half line. J. Differ. Equ. Appl..

[18] Guseinov G.S. (2002). A boundary value problem for second order nonlinear difference equations on the semi-infinite interval. Special issue in honour of Professor Allan Peterson on the occasion of his 60th birthday, Part I. J. Differ. Equ. Appl..

[19] Lian H., Li J., Agarwal R.P. (2016). Unbounded solutions of second order discrete BVPs on infinite intervals. J. Nonlinear Sci. Appl..

[20] Tian Y., Ge W. (2006). Multiple positive solutions of boundary value problems for second-order discrete equations on the half-line. J. Differ. Equ. Appl..

[21] Tian Y., Tisdell C., Ge W. (2011). The method of upper and lower solutions for discrete BVP on infinite intervals. J. Differ. Equ. Appl..

[22] Zeidler E. (1986). Nonlinear Functional Analysis and Its Applications, I Fixed-Point Theorems.

[23] Costara C., Popa D. (2003). Exercises in Functional Analysis.

[24] Migda J. (2014). Iterated remainder operator, tests for multiple convergence of series and solutions of difference equations. Adv. Differ. Equ..

[25] Migda J., Nockowska-Rosiak M. (2019). Asymptotic properties of solutions to difference equations of Sturm-Liouville type. Appl. Math. Comput..

